# Angiotensin receptor blockers and risk of dementia: cohort study in UK Clinical Practice Research Datalink

**DOI:** 10.1111/bcp.12511

**Published:** 2015-01-20

**Authors:** Kah L Goh, Krishnan Bhaskaran, Caroline Minassian, Stephen J W Evans, Liam Smeeth, Ian J Douglas

**Affiliations:** 1 KLG Drug Safety LtdWalnut House, 34 Rose Street, Wokingham, RG40 1XU, UK; 2 Department of Noncommunicable Diseases Epidemiology, London School of Hygiene and Tropical MedicineKeppel Street, London, WC1E 7HT, UK; 3 Department of Medical Statistics, London School of Hygiene and Tropical MedicineKeppel Street, London, WC1E 7HT, UK

**Keywords:** angiotensin-converting enzyme inhibitors, angiotensin receptor blockers, dementia

## Abstract

**Aims:**

This was a cohort study to evaluate whether individuals exposed to angiotensin receptor blockers have a reduced risk of dementia compared with those exposed to angiotensin-converting enzyme inhibitors.

**Methods:**

The study included new users of angiotensin receptor blockers or angiotensin-converting enzyme inhibitors (from 1995 to 2010) from UK primary care practices contributing to the Clinical Research Practice Datalink. The association between exposure to angiotensin receptor blockers and the risk of incident dementia was analysed using a Cox model, adjusting for age, sex, body mass index, diabetes, hypertension, heart failure, statin use, socioeconomic status, alcohol, smoking, number of consultations and calendar year.

**Results:**

A total of 426 089 persons were included in the primary analysis, with 45 541 persons exposed to angiotensin receptor blockers and the remainder to angiotensin-converting enzyme inhibitors. The total number of new diagnoses of dementia was 6517. There was weak evidence of a decreased risk of dementia with exposure to angiotensin receptor blockers, with follow-up beginning at 1 year after the start of treatment (adjusted hazard ratio 0.92, 95% confidence interval 0.85–1.00). An analysis restricted to the first 12 months after the index date showed a larger effect on dementia risk (adjusted hazard ratio 0.60, 95% confidence interval 0.50–0.72).

**Conclusions:**

A small reduction in dementia risk was seen with angiotensin receptor blockers in comparison to angiotensin-converting enzyme inhibitors. However, the strongest association was seen in early follow-up, suggesting that the inverse association is unlikely to be causal, but instead reflects other important but unmeasured differences between angiotensin receptor blocker and angiotensin-converting enzyme inhibitor users.

**What is Already Known about this Subject**

- Experimental studies suggest that treatments targeting the renin–angiotensin pathway could modify the risk of dementia.
- Two observational studies indicate that exposure to angiotensin II receptor blockers is associated with a decreased risk of dementia when compared with angiotensin-converting enzyme inhibitors. However, data from a large randomized clinical trial were inconclusive.

**What this Study Adds**

- There was limited evidence of an inverse association between exposure to angiotensin receptor blockers and the risk of dementia, with greater risk reduction in the first year of follow-up. This is unlikely to represent a causal association, as any beneficial drug effect is unlikely to operate on such a short time scale.

## Introduction

Dementia is characterized by a progressive decline in intellect, including memory, learning, orientation, language, comprehension and judgement 1. It is a major public health problem, with ∼36 million people worldwide estimated to be living with dementia, and the numbers are projected to double every 20 years 1. The key risk factor for most types of dementia is advanced age, although cardiovascular risk factors, such as diabetes mellitus, hypercholesterolaemia and hypertension in mid-life, also contribute 2–6. Thus, the modification of these risk factors may prevent or delay the onset of dementia.

The renin–angiotensin system (RAS) is a hormone system that regulates blood volume and systemic vascular resistance, which in turn controls cardiac output and arterial pressure. Experimental studies suggest that treatments targeting this pathway, such as angiotensin-converting enzyme inhibitors (ACEIs) and angiotensin II type 1 receptor blockers (ARBs), may have beneficial effects against the development or progression of cognitive decline and dementia 7. However, there are also some indications from animal studies that long-term treatment with ACEIs may have paradoxical neurotoxic effects 8,9, and ARBs may have greater neuroprotective effect than ACEIs through prevention of vascular damage induced by β-amyloid 10.

Clinical data comparing the effects of ARBs and ACEIs on dementia outcomes are limited. Two observational studies indicated that ARB exposure is associated with a reduced incidence of dementia when compared with ACEIs or other cardiovascular drugs 11,12. However, a blinded randomized clinical trial with telmisartan (ARB) and ramipril (ACEI) found no difference between the treatments for dementia and cognitive outcomes 13.

We used real-world data from the UK Clinical Practice Research Datalink (CPRD) to assess the overall risk of dementia associated with the use of ARBs in comparison to ACEIs. Our hypothesis was that those exposed to ARBs have a reduced risk of developing dementia compared with those exposed to ACEIs.

## Methods

### The Clinical Practice Research Datalink

The CPRD is a clinical database containing records from computer systems used by general practitioners (GPs) to record all clinical information 14,15. The database comprises anonymized computerized medical records from GPs in the UK, covering ∼8% of the UK population. The data recorded in the CPRD include demographic information, prescription details, clinical events, preventive care provided and information from secondary care settings. CPRD data collection started in 1987; there are ∼5 million patients currently registered, and 12 million patient records in total. Diagnostic accuracy for dementia recorded in the CPRD has been previously validated, where 90% of the people recorded as having dementia or Alzheimer's disease were found to have well-documented progressive dementia on detailed review of the records 16.

### Study participants

**Figure 1:**
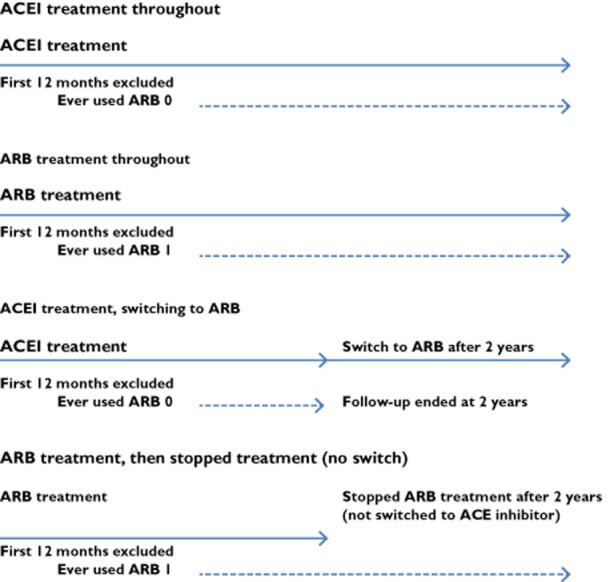
Assignment of ever exposure to ARB and the duration of follow-up using example scenarios. Abbreviations are as follows: ACEI, angiotensin-converting enzyme inhibitor; ARB, angiotensin receptor blocker

Data on all patients aged ≥18 years with a first recorded prescription for an ACEI or ARB in the years 1995–2010 inclusive and dated at least 6 months after their registration date within the CPRD were retrieved. The 6 month period ensures that the majority of those included will be new users, and excludes prevalent users with unknown previous duration of therapy. Angiotensin-converting enzyme inhibitors were defined as all drugs classified in the British National Formulary (BNF) under Chapter 2.5.5.1, and ARBs as all drugs classified under BNF Chapter 2.5.5.2.12 17 (see Tables S1 and S2). In order to prevent pre-existing, undiagnosed dementia from affecting our results, the initial 12 months of person-time following the first ARB or ACEI prescription were excluded, so individuals developing dementia or ending follow-up during this period did not contribute to our main analyses. Individuals switching from ACEI to ARB therapy or from ARB to ACEI therapy during follow-up were censored at the date of switching, as there have been suggestions that the protective affects of ARBs and ACEIs may persist for years after stopping treatment 18,19 (see Figure 1). A separate exploratory analysis was planned to examine the potential synergistic effect of combined ACEI and ARB treatment on the risk of dementia for individuals who were started on the combination treatment. However, only 193 individuals were started on the combination and so this analysis was not feasible and these individuals were excluded from the study. There are also recent papers suggesting that the combination is not advantageous and may be hazardous, at least in diabetic patients 20–22.

Individuals with records of dementia (all aetiologies) or cognitive impairment prior to the index date were excluded from the study. Individuals with a record of pre-existing dementia recorded after the index date (e.g. history of dementia, dementia annual review) without a record of when dementia was first diagnosed were also excluded from the study, as dementia may be prevalent at the start of ARB or ACEI therapy for these individuals.

### Drug exposure and outcomes data

We used ever exposure to ARBs (hereafter ‘ever ARB’) *vs*. never exposure to ARB (i.e. ACEI treatment only) as the main exposure variable, based on CPRD prescriptions data. All prescriptions for an ARB or ACEI were retrieved, and the length of each prescription was calculated based on the recorded number of tablets prescribed and the daily dose; where these data were not available, the median value (28 days) was assumed.

The outcomes of interest in this study were new diagnoses of dementia as recorded by Read Codes (see Tables S3–S5), but we excluded specific causes of dementia, which are secondary to other medical conditions and where ARBs are not expected to have any potential effect (e.g. dementia due to neoplastic disease, dementia in Creutzfeldt–Jakob disease).

### Statistical methods

Follow-up time began at the end of the 12 month qualifying period following the first prescription of either an ACEI or ARB (the index date) and ended at the earliest of the following: an incident dementia diagnosis; death; transfer out of the CPRD practice network; last data collection date for the practice; or date of treatment switch between ARB and ACEI. Otherwise, subjects remained assigned to their treatment group even if the respective treatments were stopped. Crude hazard ratios for ever exposure to ARBs *vs*. never exposure to ARB (i.e. ACEI treatment only) were calculated using Cox regression models for the outcome. Cox models were then adjusted for the following potential confounders, evaluated at the index date: age at first prescription for ARB or ACEI (18–54, 55–64, 65–74, ≥75 years), sex, body mass index (BMI) category (underweight, normal, overweight/obese), smoking status (nonsmoker, current smoker, ex-smoker), alcohol status (nondrinker, ex-drinker or current drinker, which was further classified as light, moderate, heavy, unknown), diabetes status (categorized as no evidence of diabetes, diabetes without metformin or insulin use, diabetes with metformin but no insulin use or diabetes with any insulin use), hypertension (based on a recording of diagnosed hypertension or blood pressure >140/90 mmHg), heart failure, statin use, socioeconomic status (assigned based on postcode-linkage and divided into five categories with class 1 being least socially deprived and class 5 most socially deprived), number of consultations in the preceding 6 months (≤2, 3–5, 6–9, 10–14, ≥15) and calendar year (1995–1999, 2000–2004, 2005–2010). Attained age (<60, 60–69, 70–79, ≥80 years) was also included as a time-updated covariate, because age is an important determinant of the risk of dementia.

As there is some evidence that the age at which antihypertensive treatment is initiated may have a different effect on the subsequent risk of dementia 23, a potential interaction between age at index date was investigated by adding an interaction term in the Cox regression model. The primary analysis excluded 9.2% of individuals with missing data on smoking, alcohol, BMI or socioeconomic status.

### Sensitivity analyses, model checking and other analyses

A number of planned sensitivity analyses were carried out. First, the primary analysis was repeated using attained age, rather than time since start of treatment, as the principal time scale, to provide the finest possible control for age. Second, we assessed associations with ARB use in the initial 12 months of exposure to provide further information on whether any observed associations were likely to represent a causal relationship; associations in the first 12 months would argue against this, because a causal effect would be unlikely to operate on such a short time scale. Third, we investigated the effect for a subset of individuals who received repeated prescriptions for ARBs and ACEIs for at least 1 year, because this would represent a subset of individuals who are likely to be more compliant with treatment. Fourth, for those individuals who stopped ARB or ACEI treatment but continued to have follow-up data after drug discontinuation, a sensitivity analysis censoring the follow-up 3 months after the last dose of ARB or ACEI was also conducted. In order to ascertain the likelihood of the direction of bias with the exclusion of individuals with missing data from the primary analysis, the available information on these individuals was compared with those individuals who had complete data, including the crude event rates with respect to the exposure status. Finally, the proportional hazards assumption was tested by evaluation of whether the estimated hazard ratio changes with time by splitting the time since starting treatment into a number of categories (>1 to ≤3, >3 to ≤5, >5 to ≤7, >7 years) for the ARB-exposure variable and other covariates included in the Cox model. Where there was evidence of nonproportional hazards, an interaction between the variable and treatment time categories was included to verify whether it had an effect between ARB exposure and dementia. A *post hoc* analysis was also conducted to verify whether the adjusted hazard ratio for dementia risk with ARB exposure varied with the time since starting treatment.

Two further *post hoc* exploratory analyses were conducted. First, we examined the role of specific ARB drugs (telmisartan, candesartan) that are considered to cross the blood–brain barrier and therefore potentially have activity on the central nervous system. For this analysis, patients were assumed to be exposed to only a single drug within the ARB class during follow-up, taken as the first ARB prescribed. Those treated with centrally acting ACEIs (captopril, fosinopril, perindopril, ramipril, trandolapril and lisinopril) 24, taken as the first ACEI prescribed, were considered the ‘unexposed’ individuals.

The second *post hoc* analysis was conducted with the additional adjustment of history of stroke prior to index date. Although stroke is a known risk factor for dementia 25, it was not anticipated that prior history of stroke would have an impact on the decision to prescribe ARB or ACEI for this cohort during the time of this study 26,27, hence prior history of stroke was not included in the primary analysis defined *a priori*. This additional adjustment for the history of stroke was included in the *post hoc* analysis for the risk of dementia with ARB exposure for the follow-up period starting 12 months after the index date (same follow-up period as for the primary analysis) and also in the initial 12 months after exposure to ARB or ACEI.

This study protocol was finalized prior to the start of the study and has been approved by the London School of Hygiene and Tropical Medicine Research Ethics Committee (application number: 011/286) and the Independent Scientific Advisory Committee of the Medicines and Healthcare products Regulatory Agency.

## Results

### Study population and baseline characteristics

**Figure 2:**
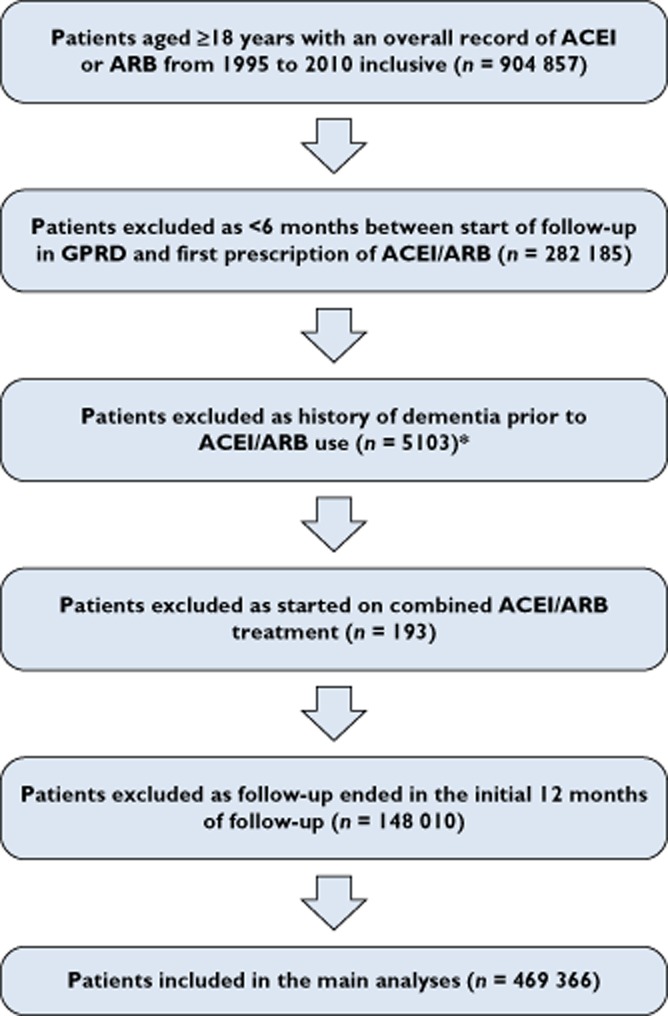
Inclusion and exclusion of study participants. Abbreviations are as in Figure 1. *Individuals with a history of dementia (*n* = 5103): ACEI users = 4761 (0.84% out of a total of 563 792 ACEI users); ARB users = 339 (0.58% out of a total of 58 684 ARB users); and ACEI+ARB users = 3 (1.53% out of a total of 196 ACEI+ARB users)

Of 904 857 patients identified with at least one ARB or ACEI prescription, 469 366 were included in the study (see Figure 2). Exclusions were mainly due to failure to meet the new user criteria (<6 months between start of follow-up in the database before first prescription; (*n* = 282 185), prior history of dementia (*n* = 5103) and follow-up ended in the initial 12 months after starting ARB or ACEI (*n* = 148 010). The median interval from the first ever ACEI or ARB prescription to the end of follow-up was 4.25 years (interquartile range 2.51–6.65), with 190 373 persons (40.6%) with follow-up ending at least 5 years after start of prescription. The proportion of follow-up covered by ARB prescription among ARB users was 0.83 and the proportion of follow-up covered by ACEI prescription among ACEI users was 0.80. There were a total of 7427 incident dementia cases recorded after the first 12 months of initiation of ARB or ACEI treatment. A total of 419 047 persons (89.3%) received ACEI treatment and 50 319 persons (10.7%) received ARB treatment.

The demographics and other baseline characteristics of all 469 366 persons by drug exposure are summarized in Table 1. Those beginning and remaining on ACEI therapy were more likely to be men (ACEI 52.9%, ARB 45.0%), have a history of diabetes (ACEI 24.4%, ARB 16.6%), heart failure (ACEI 8.1%, ARB 3.2%) and recorded statin use at baseline (ACEI 42.1%, ARB 33.9%), while hypertension (ACEI 94.7%, ARB 97.6%) was more common among those who were started on ARBs. The prescribing guidelines in use during the time of the study would generally favour ACEI treatment over ARB treatment for patients requiring blockade of the RAS 27–29.

**Table 1:** Baseline and demographic characteristics by treatment use during follow-up in people prescribed angiotensin receptor blockers (ARBs) or angiotensin-converting enzyme inhibitors (ACEIs)

Characteristic	All patients	ACEI	ARB
	(*n* = 469 366)	(*n* = 419 047)	(*n* = 50 319)
**Total observation time (million person-years)**	**1.8**	**1.6**	**0.2**
**Age (years)**			
**18–54**	118 902 (25.3)	105 962 (25.3)	12 940 (25.7)
**55–64**	116 563 (24.9)	103 708 (24.7)	12 855 (25.5)
**65–74**	119 809 (25.5)	106 785 (25.5)	13 024 (25.9)
**≥75**	114 092 (24.3)	102 592 (24.5)	11 500 (22.9)
**Sex**			
**Female**	225 038 (48.0)	197 366 (47.1)	27 672 (55.0)
**Male**	244 328 (52.0)	221 681 (52.9)	22 647 (45.0)
**Body mass index**			
**Underweight**	13 225 (2.8)	11 841 (2.8)	1 384 (2.7)
**Normal**	102 956 (21.9)	92 040 (22.0)	10 916 (21.7)
**Overweight/obese**	326 532 (69.6)	291 305 (69.5)	35 227 (70.0)
**Missing**	266 53 (5.7)	23 861 (5.7)	2 792 (5.6)
**Smoking**			
**No**	239 130 (50.9)	210 916 (50.3)	28 214 (56.1)
**Ex**	138 723 (29.6)	125 255 (29.9)	13 468 (26.8)
**Yes**	87 232 (18.6)	78 877 (18.8)	8 355 (16.6)
**Missing**	4 281 (0.9)	3 999 (1.0)	282 (0.5)
**Alcohol**			
**Nondrinker**	87 697 (18.7)	77 586 (18.5)	10 111 (20.1)
**Ex-drinker**	11 416 (2.4)	10 294 (2.5)	1 122 (2.2)
**Current low**	105 113 (22.4)	94 043 (22.4)	11 070 (22.0)
**Current medium**	8 737 (1.8)	7 777 (1.9)	960 (1.9)
**Current high**	7 081 (1.5)	6 413 (1.5)	668 (1.3)
**Current unknown amount**	219 523 (46.8)	196 519 (46.9)	23 004 (45.7)
**Missing**	29 799 (6.4)	26 415 (6.3)	3 384 (6.8)
**Hypertension**			
**Yes**	445 801 (95.0)	396 668 (94.7)	49 133 (97.6)
**Heart failure**			
**Yes**	35 353 (7.5)	33 758 (8.1)	1 595 (3.2)
**Diabetes**			
**No**	358 623 (76.4)	316 640 (75.6)	41 983 (83.4)
**Yes – no metformin/insulin**	52 743 (11.2)	48 573 (11.6)	4 170 (8.3)
**Yes – metformin**	41 802 (8.9)	38 659 (9.2)	3 143 (6.3)
**Yes – insulin**	16 198 (3.5)	15 175 (3.6)	1 023 (2.0)
**Statin use**			
**Yes**	193 360 (41.2)	176 308 (42.1)	17 052 (33.9)
**Stroke**			
**Yes**	29 863 (7.0)	27 438 (7.2)	2 425 (5.3)
**Socioeconomic status**			
**1**	87 140 (18.6)	77 784 (18.5)	9 356 (18.6)
**2**	91 303 (19.4)	81 789 (19.5)	9 514 (18.9)
**3**	93 057 (19.8)	82 997 (19.8)	10 060 (20.0)
**4**	99 072 (21.1)	87 928 (21.0)	11 144 (22.1)
**5**	98 580 (21.0)	88 343 (21.1)	10 237 (20.3)
**Missing**	214 (0.1)	206 (0.1)	8 (0.1)
**Calendar year**			
**1995–1999**	60 083 (12.8)	56 185 (13.4)	3 898 (7.8)
**2000–2004**	185 476 (39.5)	159 405 (38.0)	26 071 (51.8)
**2005–2010**	223 807 (47.7)	203 457 (48.6)	20 350 (40.4)

Figures are numbers (percentages) unless stated otherwise. *P* Values are from χ^2^ test for categorical variable. Statistical tests comparing the ACEI *vs*. ARB groups gave strong evidence of differing distributions (*P* < 0.001) of all variables in the table except body mass index (where *P* = 0.13).

There were missing data on BMI (5.7%), smoking status (0.9%), alcohol status (6.9%) and SES (0.1%); 426 089 persons (90.8%) had complete data for all baseline variables.

### Effect of ARB ever exposure (primary analysis)

After excluding patients with missing data (*n* = 43 277), a total of 426 089 persons were included in the primary analysis comparing the risk of dementia in the ever ARB exposed (*n* = 45 541) with the unexposed (*n* = 380 548; i.e. those exposed to ACEI), with the observation time starting at 1 year after the index date. The total number of recorded events of dementia (excluding dementia secondary to other medical conditions where ARB is not expected to have an effect) was 6517.

**Figure 3:**
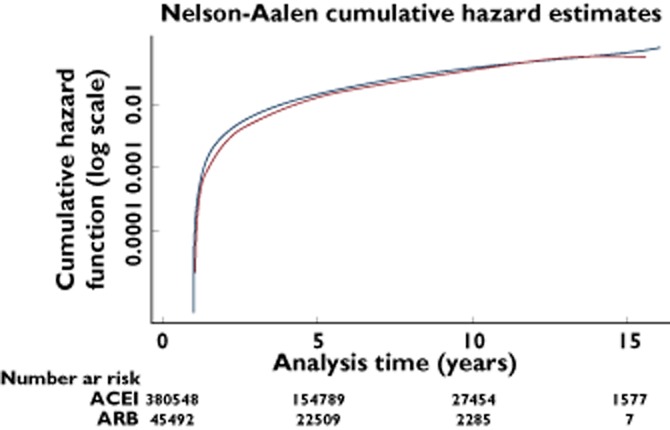
Survival curve for incident dementia in study cohorts included in the primary analysis. Abbreviations are as in Figure 1. Cox regression model with ever exposure to ARB, adjusted for age at first prescription, sex, body mass index, smoking, alcohol use, diabetes, hypertension, heart failure, statin use, socioeconomic status, calendar year, number of consultations in the last 6 months and attained age (time-updated variable). Follow-up started 1 year after first exposure to ARB or ACEI. —, ACEI; —, ARB

The crude incidence rate of dementia was lower in the ARB group [3.46 events per 1000 person-years, 95% confidence interval (CI) 3.20–3.73] compared with the ACEI group (3.91 events per 1000 person-years, 95% CI 3.81–4.01), with the crude hazard ratio of 0.89 (95% CI 0.82–0.97; see Table 2). After adjusting for potential confounders, there was weak evidence of an inverse association between ever ARB exposure and incident diagnosis of dementia (adjusted hazard ratio 0.92, 95% CI 0.85–1.00, *P* = 0.04). Figure 3 shows the survival curve for the association between ever ARB exposure and the risk of dementia over treatment time. The curves appear to be furthest apart during early follow-up and tend to come together over time. There was little indication of an interaction between the age at index date and ARB exposure in Cox regression analysis (adjusted hazard ratio 0.88, 95% CI 0.35–2.21, *P* = 0.43).

**Table 2:** Primary analysis for incidence rates of dementia by treatment and crude and adjusted hazard ratios in people taking angiotensin receptor blockers or angiotensin-converting enzyme inhibitors

ARB exposure	Total number of new diagnoses of dementia	Total person-time (×10^5^ person-years)	Incidence rate (per 10^3^ person-years)	Crude HR (95% CI)	*P* Value	Adjusted HR* (95% CI)	*P* Value
**ARB exposure**							
**ACEI use only**	5853	14.96	3.91 (3.81–4.01)	1.0		1.0	
**Ever ARB use**	664	1.92	3.46 (3.20–3.73)	0.89 (0.82–0.97)	0.004	0.92 (0.85–1.00)	0.04
**Age at first prescription (years)**							
**18–54**	51	4.46	0.11 (0.09–0.15)	1.0		1.0	
**55–64**	331	4.54	0.73 (0.65–0.81)	6.39 (4.76–8.58)		2.83 (1.86–4.30)	
**65–74**	1949	4.60	4.24 (4.06–4.43)	37.5 (28.38–49.48)		6.03 (3.87–9.40)	
**≥75**	4186	3.28	12.77 (12.39–13.16)	118.72 (90.07–156.49)	<0.001	9.53 (6.04–15.03)	<0.001
**Gender**							
**Female**	4015	8.01	5.01 (4.86–5.17)	1.0		1.0	
**Male**	2502	8.86	2.82 (2.71–2.94)	0.56 (0.54–0.59)	<0.001	0.82 (0.78–0.87)	<0.001
**Body mass index**							
**Underweight**	497	0.43	11.43 (10.47–12.48)	1.0		1.0	
**Normal**	2367	3.89	6.08 (5.84–6.33)	0.52 (0.48–0.58)		0.73 (0.66–0.81)	
**Overweight**	3653	12.55	2.91(2.82–3.01)	0.25 (0.23–0.27)	<0.001	0.51 (0.47–0.56)	<0.001
**Smoking**							
**No**	3728	8.78	4.25 (4.11–4.39)	1.0		1.0	
**Ex**	1995	4.96	4.02 (3.85–4.20)	0.96 (0.91–1.01)		1.03 (0.98–1.09)	
**Current**	794	3.14	2.53 (2.36–2.71)	0.60 (0.56–0.65)	<0.001	1.03 (0.95–1.12)	0.48
**Alcohol**							
**No**	1903	3.30	5.76 (5.11–6.03)	1.0		1.0	
**Ex**	227	0.40	5.67 (4.98–6.46)	1.00 (0.87–1.15)		1.25 (1.09–1.44)	
**Current low**	1648	4.00	4.12 (3.93–4.32)	0.71 (0.67–0.76)		0.89 (0.83–0.95)	
**Current medium**	73	0.35	2.11 (1.67–2.65)	0.36 (0.29–0.46)		0.78 (0.62–0.99)	
**Current high**	51	0.23	2.22 (1.68–2.92)	0.39 (0.29–0.51)		1.22 (0.92–1.61)	
**Current unknown amount**	2615	8.60	3.04 (2.93–3.16)	0.53 (0.50–0.56)	<0.001	0.85 (0.79–0.90)	<0.001
**Hypertension**							
**No**	265	0.84	3.14 (2.79–3.55)	1.0		1.0	
**Yes**	6252	16.03	3.90 (3.80–4.00)	1.27 (1.12–1.43)	<0.001	0.95 (0.84–1.07)	0.39
**Heart failure**							
**No**	5583	15.78	3.54 (3.45–3.63)	1.0		1.0	
**Yes**	934	1.11	8.42 (7.90–8.98)	2.37 (2.22–2.54)	<0.001	1.15 (1.07–1.23)	<0.001
**Diabetes**							
**No**	4816	12.85	3.75 (3.64–3.86)	1.0		1.0	
**Yes – no metformin/insulin**	992	1.91	5.19 (4.88–5.53)	1.40 (1.31–1.50)		1.11 (1.03–1.19)	
**Yes – metformin**	525	1.50	3.50 (3.22–3.82)	0.95 (0.87–1.04)		1.17 (1.07–1.29)	
**Yes – insulin**	184	0.62	2.99 (2.58–3.45)	0.80 (0.69–0.92)	<0.001	1.18 (1.02–1.38)	<0.001
**Statin use**							
**No**	4064	10.48	3.88 (3.76–4.00)	1.0		1.0	
**Yes**	2453	6.39	3.84 (3.69–3.99)	1.03 (0.98–1.08)	0.30	1.03 (0.98–1.09)	0.26
**Socioeconomic status**							
**1**	1245	3.18	3.92 (3.71–4.14)	1.0		1.0	
**2**	1230	3.29	3.74 (3.53–3.95)	0.96 (0.88–1.03)		0.97 (0.89–1.05)	
**3**	1321	3.24	4.07 (3.86–4.30)	1.05 (0.97–1.13)		1.00 (0.92–1.08)	
**4**	1332	3.57	3.74 (3.54–3.94)	0.96 (0.89–1.03)		0.94 (0.87–1.02)	
**5**	1389	3.60	3.86 (3.66–4.07)	0.99 (0.91–1.06)	0.13	1.09 (1.01–1.18)	0.003
**Calendar year**							
**1995–1999**	1412	3.62	3.90 (3.70–4.10)	1.0		1.0	
**2000–2004**	3525	8.60	4.10 (3.97–4.24)	1.16 (1.09–1.24)		1.13 (1.05–1.21)	
**2005–2010**	1580	4.66	3.39 (3.23–3.57)	1.07 (0.98–1.16)	<0.001	1.14 (1.04–1.24)	0.002
**No. of consultations**							
**≤2**	224	1.11	2.01 (1.77–2.30)	1.0		1.0	
**3–5**	824	3.33	2.47 (2.31–2.65)	1.22 (1.06–1.42)		1.05 (0.90–1.21)	
**6–9**	1843	5.25	3.51 (3.36–3.68)	1.74 (1.51–1.99)		1.15 (1.00–1.32)	
**10–14**	1862	4.23	4.40 (4.21–4.61)	2.19 (1.91–2.52)		1.20 (1.05–1.38)	
**≥15**	1764	2.96	5.97 (5.70–6.25)	3.02 (2.63–3.47)	<0.001	1.42 (1.24–1.64)	<0.001
**Attained age (time-updated variable; years)**							
**<60**	65	5.01	0.13 (0.10–0.17)	1.0		1.0	
**≥60**	358	4.58	0.78 (0.70–0.87)	6.00 (4.61–7.82)		2.20 (1.50–3.21)	
**≥70**	2154	4.53	4.75 (4.56–4.96)	36.47 (28.50–46.68)		6.40 (4.28–9.57)	
**≥80**	3940	2.76	14.27 (13.83–14.72)	109.32 (85.55–139.72)	<0.001	11.90 (7.86–18.01)	<0.001

* Adjusted for age at first prescription, sex, body mass index, smoking, alcohol use, diabetes, hypertension, heart failure, statin use, socioeconomic status, calendar year, number of consultations in last 6 months and attained age (time-updated variable). The likelihood ratio test was used for significance testing. Abbreviations are as follows: ACEI, angiotensin-converting enzyme inhibitor; ARB, angiotensin receptor blocker; CI, confidence interval; HR, hazard ratio.

### Sensitivity analyses

The results of the sensitivity analyses are summarized in Table 3. When the analysis was repeated using age as the primary time scale rather than treatment time, the estimate for the hazard ratio for dementia was unchanged (adjusted hazard ratio 0.92, 95% CI 0.85–1.00). There was evidence that ever ARB exposure was associated with a large reduction on the risk of dementia within the first year of starting treatment (hazard ratio 0.60, 95% CI 0.50–0.72, *P* < 0.001).

**Table 3:** Sensitivity and exploratory analyses: incidence rates of dementia by treatment and crude and adjusted hazard ratios in people taking angiotensin receptor blockers or angiotensin-converting enzyme inhibitors

ARB exposure	Total number of new diagnoses of dementia	Total person-time (×10^5^ person-years)	Incidence rate (per 10^3^ person-years)	Crude HR (95% CI)	*P* Value	Adjusted HR* (95% CI)	*P* Value
**Start of follow-up at 12 months after first prescription (attained age as time scale)**							
**ACEI use only**	5853	14.96	3.91 (3.81–4.01)	1.0		1.0	
**Ever ARB use**	664	1.92	3.46 (3.20–3.73)	0.92 (0.85–0.99)	0.03	0.92 (0.85–1.00)†	0.04
**Start of follow-up from treatment initiation up to the initial 12 months**							
**ACEI use only**	1880	4.32	4.35 (4.16–4.55)	1.0		1.0	
**Ever ARB use**	127	0.49	2.60 (2.19–3.10)	0.60 (0.50–0.72)	<0.001	0.60 (0.50–0.72)	<0.001
**Start of follow-up at 12 months after first prescription (time since starting treatment as time scale)**							
**Prescription of ARB/ACEI for at least 1 year**							
**ACEI use only**	4171	11.54	3.61 (3.51–3.73)	1.0		1.0	
**Ever ARB use**	511	1.57	3.24 (2.97–3.53)	0.90 (0.82–0.99)	0.04	0.91 (0.83–1.00)	0.05
**Follow-up ending at 3 months after last dose in those who stopped ACEI or ARB**							
**ACEI use only**	4524	12.50	3.62 (3.51–3.73)	1.0		1.0	
**Ever ARB use**	518	1.60	3.23 (2.97–3.52)	0.88 (0.80–0.96)	0.004	0.91 (0.83–0.99)	0.03
**Exploratory analysis**							
**Centrally acting ARBs *vs*. ACEIs (based on first prescription)**							
**ACEI use only**	5267	13.33	3.95 (3.84– 4.06)	1.0		1.0	
**Ever ARB use**	142	0.43	3.34 (2.83–3.93)	0.85 (0.72–1.01)	0.06	0.84 (0.71–1.00)	0.04

* Adjusted for age at first prescription, sex, body mass index, smoking, alcohol use, diabetes, hypertension, heart failure, statin use, socioeconomic status, calendar year, number of consultations in the last 6 months and attained age (time-updated variable). †Adjusted for age at first prescription, sex, body mass index, smoking, alcohol use, diabetes, hypertension, heart failure, statin use, socioeconomic status, calendar year, number of consultations in the last 6 months and time since starting treatment (time-updated variable). The likelihood ratio test was used for significance testing. Abbreviations are as for Table 2.

In the analysis based on a subset of patients who had received at least 1 year of prescriptions for ARB or ACEI, a total of 323 197 individuals remained at risk after the first year of treatment. The estimated hazard ratio for this subset of patients was similar (adjusted hazard ratio 0.91, 95% CI 0.83–1.00) to the results for all ever ARB exposed. In the analysis restricted to users of centrally acting agents only, the estimated hazard ratio suggested a greater reduction in dementia risk (adjusted hazard ratio 0.84, 95% CI 0.71–1.00), but this is clearly limited evidence for any real difference given the wide confidence interval.

A sensitivity analysis censoring the follow-up period to up to 3 months after the last dose of ARB or ACEI for individuals who stopped ARB or ACEI treatment showed similar estimates for the hazard ratio (adjusted hazard ratio 0.91, 95% CI 0.83–0.99).

Inclusion of history of stroke as an additional covariate (to the original model with adjustment for age at first prescription for ARB or ACEI, sex, BMI category, smoking status, alcohol status, diabetes status, hypertension, heart failure, statin use, socioeconomic status, number of consultations in the preceding 6 months, calendar year and attained age) in a sensitivity analysis did not alter the findings from the primary analysis. The adjusted hazard ratio for dementia with additional adjustment for history of stroke was 0.93 (95% CI 0.85–1.01) with follow-up starting at 12 months after the index date. With the repeat analysis of the risk of dementia with ARB exposure in the initial 12 months following the index date with the additional adjustment for stroke, the adjusted hazard ratio for dementia was 0.61 (95% CI 0.51–0.73).

For 9.2% of the individuals with missing data for BMI, alcohol use, smoking or socioeconomic status, the crude incidence rate of dementia was found to be higher (6.68 events per 1000 person-years, 95% CI 6.26–7.13) when compared with those who had complete data (3.86 events per 1000 person-years, 95% CI 3.77–3.96). However, there were differences in some of the baseline characteristics and risk factors for dementia between the two groups of individuals; there was a higher proportion of those aged ≥75 years (missing 40.6%, complete 22.7%), female patients (missing 52.2%, complete 47.5%), with history of heart failure (missing 14.4%, complete 6.8%), but a lower proportion with a history of diabetes (missing 13.7%, complete 24.6%), hypertension (missing 88.8%, complete 95.6%) and recorded use of statin at baseline (missing 28.6%, complete 42.5%) for the individuals with missing data (see Table S6). Among those with missing data, the crude incidence rate of dementia for ACEI users was 7.00 per 1000 person-years (95% CI 6.54–7.50) compared with 4.50 per 1000 person-years in ARB users (95% CI 3.60–5.61). Of note, there was a marked difference in the proportion of individuals aged ≥75 years between ACEI and ARB users (ACEI 41.4%, ARB 33.6%) for those with missing data, in contrast to the individuals with complete data (ACEI 22.8%, ARB, 21.7%; see Table S7).

### Model checking

Evaluation of the Cox model with the inclusion of all covariates has shown evidence of apparent nonproportional hazards for the ARB exposure variable (*P* = 0.02). In a *post hoc* analysis, stratifying the treatment effect by time since start of treatment has shown an adjusted hazard ratio of 0.83 (95% CI 0.72–0.96) for the follow-up interval of >1 to ≤3 years, 0.87 (95% CI 0.75–1.01) for >3 to ≤5 years, 1.07 (95% CI 0.90–1.29) for >5 to ≤7 years and 1.05 (95% CI 0.86–1.29) for >7 years (see Table 4). There was also evidence of nonproportional hazards for the covariate of calendar period (*P* = 0.03), but the inclusion of an interaction between follow-up time and calendar period did not alter the estimated association between ARB exposure and dementia (hazard ratio 0.92, 95% CI 0.85–1.00).

**Table 4:** Interaction between time since starting treatment and ever exposure to angiotensin receptor blockers on the risk of dementia

Time after starting treatment	Adjusted HR* (95% CI)	*P* Value
**>1 to ≤3 years**	0.83 (0.72–0.96)	0.009
**>3 to ≤5 years**	0.87 (0.75–1.01)	0.06
**>5 to ≤7 years**	1.07 (0.90–1.29)	0.44
**>7 years**	1.05 (0.86–1.29)	0.48

* Adjusted for age at first prescription, sex, body mass index, smoking, alcohol use, diabetes, hypertension, heart failure, statin use, socioeconomic status, calendar year, number of consultations in the last 6 months and attained age (time-updated variable). The likelihood ratio test was used for significance testing.

Abbreviations are as follows:

CI, confidence interval;

HR, hazard ratio.

## Discussion

### Summary of main findings

In this large cohort of new users of ACEIs or ARBs, we found limited evidence of reduced risk of incident dementia in users of ARBs compared with ACEIs (adjusted hazard ratio 0.92, 95% CI 0.85–1.00). A *post hoc* analysis has shown evidence of interaction between follow-up time with ever exposure to ARB, such that an inverse association with a reduction in risk for dementia was found in the early follow-up period, but not observed with longer follow-up. A separate analysis has also shown that the risk reduction appears greatest during the first year of treatment (adjusted hazard ratio 0.60, 95% CI 0.50–0.72).

The findings are contrary to expectations based on current knowledge, which suggests that ARBs may prevent or delay the onset of dementia via remodelling of the systemic vasculature or brain microvasculature 30,31. Such a mechanism would suggest that any risk reduction would be greater with a longer exposure time, because the neuropathological changes associated with dementia precede the clinical onset of disease by many years 32,33. It is unlikely that a pharmacological agent could reverse these chronic changes within a short period of 1 year to produce such a dramatic clinical effect. We therefore suggest that the inverse association between dementia risk and exposure to ARBs is likely to be explained by noncausal factors. Although ARBs and ACEIs have very similar treatment indications, the possibility of confounding by indication could not be excluded. In clinical practice, most patients are usually started on an ACEI if RAS blockade is indicated, particularly in the early years of the study 26–28,34. First, there was a stronger body of clinical evidence for ACEI compared with ARB at the time with respect to cardiovascular mortality and morbidity. Secondly, the generic forms for some ACEIs became available in the late 1990s 35,36, which were therefore less costly compared with ARBs, where generic forms for one specific ARB became available only in 2008/2009 37,38. Angiotensin receptor blockers tended to be prescribed when an ACEI was not tolerated, most commonly due to symptoms of ACEI-related cough 39,40. Comparison of the baseline characteristics of the study population showed a higher proportion of patients with diabetes, heart failure and statin use in the ACEI group than the ARB group, which is also suggestive of differences in the prescribing practices, whereby people with more comorbidities may be prescribed ACEI preferentially. Adjustment for these variables (diabetes, heart failure and statin use) was included in the primary analysis, and in a *post hoc* sensitivity analysis, history of stroke was included as an additional covariate, and the estimated hazard ratios for dementia were similar with or without the inclusion of history of stroke, suggesting that although stroke was a risk factor it was not a confounder. There may be residual confounding that could explain a difference in the risk of dementia risk observed in the first year of follow-up. For example, some variables may not be quantified sufficiently in this study, such as hypertension, which was categorized into presence/absence of hypertension, which may not be sufficiently granular. The binary classification of statin use at study baseline also does not take into account the dose of statin used and the subsequent use of statin over the course of the study, though the effect of statin use on the risk of dementia remains unclear 41,42. Indirect evidence suggesting a noncausal association was found in the analysis restricted to patients receiving regular prescriptions for at least the first year after the index date. A causal effect would tend to be stronger in this subgroup, but the reduction in risk of dementia was very similar to that found in the primary analysis, suggesting that the differences between ARB and ACEI users are most probably due to other factors. Given the large number of important differences in the baseline characteristics of the ARB and ACEI users, it is therefore likely that there may be other unmeasured confounders.

An exploratory analysis with ARBs thought to have the ability to affect the brain RAS suggests a possible larger reduction in the risk of dementia (adjusted hazard ratio 0.84, 95% CI 0.71–1.00). However, the confidence intervals for this estimate are wide, and thus, there is limited evidence of a real difference.

### Strengths and limitations

Our study included a large number of patients in a real-world clinical setting and with relatively long follow-up period. Approximately a quarter of the individuals included in the study were under 55 years old, whilst past studies on dementia have been criticized for inclusion of patients only over the age of 60 years. Given that neurodegenerative changes may precede clinical dementia by decades, it is suggested that middle-aged patients should also be included in studies investigating potential preventative treatments for dementia 33,43. Patients from the CPRD are broadly representative of the adult population in UK. The use of routinely collected data allowed for adjustment in the analyses for multiple important confounders, and the diagnostic accuracy of dementia recording has been shown to be high 16.

It has been suggested that ARBs may have a greater effect on certain subtypes of dementia (e.g. Alzheimer's disease, vascular dementia) 12,44, but this has not been demonstrated consistently 11. While we have excluded certain conditions where dementia is secondary to other medical conditions (e.g. neoplastic disease, human immunodeficiency viral disease), we did not attempt to subcategorize the recorded dementia outcomes further into disease subtypes for a number of reasons. First, the underlying pathologies for dementia can be difficult to distinguish on clinical grounds 33,45, and in postmortem studies, mixed pathology for dementia was found to be more common than ‘pure’ disease aetiology 46. Second, records of dementia in the CPRD data were very frequently nonspecific (e.g. senile dementia, unspecified dementia), and thus, the suspected disease aetiology cannot be specified accurately.

There is also the possibility of misclassification with respect to the time of onset of dementia. Given that dementia is of an insidious onset and there could be variability in the timing of patient presentation to the GP, it is often difficult to determine the actual date of disease onset. Moreover, GPs may vary in terms of the practice of recording event dates. However, there is no reason to suspect that there would be any systematic differences in the practice of recording of diagnosis dates between ARB or ACEI users, and therefore, this may not be an important source of bias for the study.

A further limitation is that we had no direct data on adherence to treatment. We used records of prescriptions to define exposure, and some patients might not have taken their drugs regularly, resulting in misclassification and potential bias in either direction.

We included only individuals with complete data in the primary analysis and excluded 9.2% of individuals with any missing data. Although the crude incidence rate of dementia in individuals with missing data for BMI, alcohol, smoking or socioeconomic status was higher than for the individuals with complete data, there was also a large difference in the proportion of individuals aged ≥75 years (missing 40.6%, complete 22.7%) between the two groups. While there were also other differences in the baseline risk factors for dementia, the difference in age distribution is likely to have a greater impact on the incidence rates, because age is a major risk factor for dementia. There was a larger difference in the crude incidence rates of dementia between ACEI and ARB users for individuals with missing data when compared with those who had complete data, but a marked difference in the proportion of individuals aged ≥75 years between ACEI and ARB users (ACEI 41.4%, ARB 33.6%) was also noted for those with missing data. There are no *a priori* reasons to suggest that the relationship between the exposure and outcome is different between those with complete data *vs*. missing data, conditional on the covariates. When missingness is independent of the outcome given the covariates, any bias resulting from complete case analysis is likely to be negligible 47.

There was evidence of nonproportionality hazards for ARB exposure. It is, however, not statistically possible to distinguish between nonproportionality of hazards and unmeasured risk factors.

### Comparison with other studies

There are short-term open-label studies (up to 24 weeks) with blinded end-points, which have shown that treatment with ARBs resulted in improvement of some measures of cognitive function tests, when compared with ACEIs. In these studies, patients treated with ARBs were found to have a greater reduction in blood pressure, which raised a question regarding whether ARB may have a greater neuroprotective effect than ACEI via blood-pressure-lowering effects 48,49.

A large randomized clinical trial, ‘The Ongoing Telmisartan Alone and in Combination with Ramipril Global Endpoint Trial (ONTARGET)’, investigated the effect of a specific ARB (telmisartan), ACEI (ramipril) and the combination of the two treatments on the clinical diagnosis of dementia and cognitive test results in 25 260 patients aged ≥55 years with established cardiovascular diseases or diabetes over median follow-up period of 56 months 13,18. Telmisartan and combined telmisartan–ramipril treatment resulted in greater reduction in blood pressure than ramipril monotherapy. The study, however, provided limited evidence that telmisartan may be superior to ramipril (odds ratio 0.90, 95% CI 0.80–1.01) with regard to cognitive impairment, but there was no evidence of an effect (odds ratio 0.97, 95% CI 0.89–1.06) on cognitive decline. Combined telmisartan and ramipril did not appear to have an impact on cognitive impairment or decline when compared with ramipril alone.

Two large observational studies investigated the association between ARBs and dementia risk, where ACEIs were included as a comparator, but these studies excluded middle-aged patients. One used data from the administrative database of the US Veteran Affairs, with individuals (predominantly male) aged 65 years or more with cardiovascular disease 11, and found that ARB use was associated with a large reduction in the incidence and progression of Alzheimer's disease and other forms of dementia when compared with a selected ACEI (lisinopril; adjusted hazard ratio for Alzheimer's disease 0.81, 95% CI 0.68–0.96; and adjusted hazard ratio for other dementia 0.81, 95% CI 0.73–0.90). However, the analysis included the first 12 months after initiation of drug and had a relatively short follow-up period (maximum 4 years).

A nested case–control study using CPRD data on patients aged ≥60 years who were ever treated with an antihypertensive investigated the relationship between exposure to ARB, ACEI, other antihypertensives and dementia risk 12. The odd ratios for probable Alzheimer's disease and any dementia (combined dementia outcomes) for the ARB group were 0.47 (95% CI 0.37–0.58) and 0.55 (95% CI 0.49–0.62), respectively, suggesting a dramatic protective effect and again raising the possibility of a noncausal explanation. These large effects have essentially been ruled out by the randomized data and so are likely to be artefactual.

## Conclusion and recommendations

We found limited evidence of a reduced risk of dementia amongst ARB compared with ACEI users. The effect was more apparent during early follow-up and diminished over time. Based on our current understanding of the pathology underlying the disease, it is unlikely that this represents a causal association, and the pattern of results seen suggests that this apparent risk reduction is explained by other underlying differences between ARB and ACEI users.

A randomized controlled trial may be the best study design to evaluate this question further, because further observational studies are likely to have similar problems with confounding. However, any clinical trial would need to be powered adequately and of sufficiently long duration to study dementia or cognitive outcomes as primary end-points.

## Competing Interests

All authors have completed the Unified Competing Interest form at http://www.icmje.org/coi_disclosure.pdf (available on request from the corresponding author) and declare: KLG received support (student loan) from Pietrek Associates Ltd during the conduct of the study and was later employed by Pietrek Associates Ltd (unrelated to the submitted work). After the submission of this work, KLG has provided contract services for Takeda Pharmaceuticals (unrelated to the submitted work). KB has received grants from National Institute for Health Research during the conduct of the study. CM has received grants from Wellcome Trust. LS has received grants from Wellcome Trust during the conduct of the study; grants from Wellcome Trust, grants from Medical Research Council, grants from National Institute of Health Research, personal fees from GlaxoSmithKline, outside the submitted work. IJD has received grants from Medical Research Council during the conduct of the study; personal fees from GlaxoSmithKline, Takeda, Gilead and holds stocks in GlaxoSmithKline, outside the submitted work. There are no other relationships or activities that could appear to have influenced the submitted work.

## 

*The data used for this study were derived from a research project on angiotensin II type I receptor blockers and the risk of cancer, which was supported by the National Institute of Health Research.*

## Supporting Information

Table S1 List of angiotensin-converting enzyme inhibitors included in the study Table S2 List of angiotensin II receptor blockers included in the study Table S3 Read codes representing dementia outcomes of interest in the study Table S4 Read codes for dementia subtypes where angiotensin receptor blockers are not expected to have an effect Table S5 Read codes indicative of pre-existing dementia or cognitive impairment Table S6 Comparison of key baseline characteristics and crude incident rates of dementia for individuals with complete data *vs*. individuals with any missing data. Figures are numbers (percentages) unless stated otherwise Table S7 Comparison of key baseline characteristics and crude incident rates of dementia by exposure status for individuals with complete data *vs*. individuals with any missing data. Figures are numbers (percentages) unless stated otherwise Table S8 *Post hoc* analysis with additional adjustment for history of stroke: incidence rates of dementia by treatment and crude and adjusted hazard ratios in people taking angiotensin receptor blockers (ARBs) or angiotensin-converting enzyme inhibitors (ACEIs) Table S9 *Post hoc* analyses: incidence rates of dementia by treatment and adjusted hazard ratios in people taking angiotensin receptor blockers (ARB) or angiotensin-converting enzyme inhibitors (ACEI), with and without additional adjustment for history of stroke
