# Supplementary material for: Angiotensin receptor blockers and risk of dementia: cohort study in UK Clinical Practice Research Datalink
Source: Br J Clin Pharmacol. 2015 Jan 20;79(2):337–50. doi: 10.1111/bcp.12511 (PMC4309639; doi:10.1111/bcp.12511)
Supplement: Supplementary file 1 — Table S1 List of angiotensin-converting enzyme inhibitors included in the study Table S2 List of angiotensin II receptor blockers included in the study Table S3 Read codes representing dementia outcomes of interest in the study Table S4 Read codes for dementia subtypes where angiotensin receptor blockers are not expected to have an effect Table S5 Read codes indicative of pre-existing dementia or cognitive impairment Table S6 Comparison of key baseline characteristics and crude incident rates of dementia for individuals with complete data vs. individuals with any missing data. Figures are numbers (percentages) unless stated otherwise Table S7 Comparison of key baseline characteristics and crude incident rates of dementia by exposure status for individuals with complete data vs. individuals with any missing data. Figures are numbers (percentages) unless stated otherwise Table S8 Post hoc analysis with additional adjustment for history of stroke: incidence rates of dementia by treatment and crude and adjusted hazard ratios in people taking angiotensin receptor blockers (ARBs) or angiotensin-converting enzyme inhibitors (ACEIs) Table S9 Post hoc analyses: incidence rates of dementia by treatment and adjusted hazard ratios in people taking angiotensin receptor blockers (ARB) or angiotensin-converting enzyme inhibitors (ACEI), with and without additional adjustment for history of stroke [file bcp0079-0337-sd1.zip › bcp12511-supp-0001-tableS3.docx]

**Supplementary information**

**Table S3 Read codes representing dementia outcomes of interest in the study**

| **Read code** | **Read Term** |
| --- | --- |
| E00..12 | Senile/presenile dementia |
| E00..11 | Senile dementia |
| F110.00 | Alzheimer's disease |
| Eu02z14 | [X] Senile dementia NOS |
| Eu02z00 | [X] Unspecified dementia |
| Eu01.00 | [X]Vascular dementia |
| E000.00 | Uncomplicated senile dementia |
| F116.00 | Lewy body disease |
| Eu00.00 | [X]Dementia in Alzheimer's disease |
| Eu00z11 | [X]Alzheimer's dementia unspec |
| E004.11 | Multi infarct dementia |
| Eu01200 | [X]Subcortical vascular dementia |
| Eu02300 | [X]Dementia in Parkinson's disease |
| Eu01.11 | [X]Arteriosclerotic dementia |
| F111.00 | Pick's disease |
| Eu01100 | [X]Multi-infarct dementia |
| Eu00112 | [X]Senile dementia,Alzheimer's type |
| Eu02.00 | [X]Dementia in other diseases classified elsewhere |
| E001.00 | Presenile dementia |
| F110000 | Alzheimer's disease with early onset |
| E002000 | Senile dementia with paranoia |
| Eu01z00 | [X]Vascular dementia, unspecified |
| E004.00 | Arteriosclerotic dementia |
| E002100 | Senile dementia with depression |
| E041.00 | Dementia in conditions EC |
| Eu00011 | [X]Presenile dementia,Alzheimer's type |
| Eu02500 | [X]Lewy body dementia |
| E001300 | Presenile dementia with depression |
| Eu02z16 | [X] Senile dementia, depressed or paranoid type |
| Eu02000 | [X]Dementia in Pick's disease |
| Eu00z00 | [X]Dementia in Alzheimer's disease, unspecified |
| E001200 | Presenile dementia with paranoia |
| Eu00200 | [X]Dementia in Alzheimer's dis, atypical or mixed type |
| Eu01300 | [X]Mixed cortical and subcortical vascular dementia |
| F110100 | Alzheimer's disease with late onset |
| Eu02z13 | [X] Primary degenerative dementia NOS |
| Eu02200 | [X]Dementia in Huntington's disease |
| E003.00 | Senile dementia with delirium |
| E001z00 | Presenile dementia NOS |
| Eu00100 | [X]Dementia in Alzheimer's disease with late onset |
| E002z00 | Senile dementia with depressive or paranoid features NOS |
| E004z00 | Arteriosclerotic dementia NOS |
| E001000 | Uncomplicated presenile dementia |
| E004000 | Uncomplicated arteriosclerotic dementia |
| E004300 | Arteriosclerotic dementia with depression |
| Eu00113 | [X]Primary degen dementia of Alzheimer's type, senile onset |
| E002.00 | Senile dementia with depressive or paranoid features |
| Eu01000 | [X]Vascular dementia of acute onset |
| Eu00111 | [X]Alzheimer's disease type 1 |
| Eu02z11 | [X] Presenile dementia NOS |
| Eu00000 | [X]Dementia in Alzheimer's disease with early onset |
| E001100 | Presenile dementia with delirium |
| Eu04100 | [X]Delirium superimposed on dementia |
| ZS7C500 | Language disorder of dementia |
| Eu01y00 | [X]Other vascular dementia |
| E004200 | Arteriosclerotic dementia with paranoia |
| Eu01111 | [X]Predominantly cortical dementia |
| E004100 | Arteriosclerotic dementia with delirium |
| Fyu3000 | [X]Other Alzheimer's disease |
| Eu00012 | [X]Primary degen dementia, Alzheimer's type, presenile onset |
| Eu00013 | [X]Alzheimer's disease type 2 |
| E02y100 | Drug-induced dementia |
| Eu02y00 | [X]Dementia in other specified diseases classif elsewhere |
