# Supplementary material for: Angiotensin receptor blockers and risk of dementia: cohort study in UK Clinical Practice Research Datalink
Source: Br J Clin Pharmacol. 2015 Jan 20;79(2):337–50. doi: 10.1111/bcp.12511 (PMC4309639; doi:10.1111/bcp.12511)
Supplement: Supplementary file 1 — Table S1 List of angiotensin-converting enzyme inhibitors included in the study Table S2 List of angiotensin II receptor blockers included in the study Table S3 Read codes representing dementia outcomes of interest in the study Table S4 Read codes for dementia subtypes where angiotensin receptor blockers are not expected to have an effect Table S5 Read codes indicative of pre-existing dementia or cognitive impairment Table S6 Comparison of key baseline characteristics and crude incident rates of dementia for individuals with complete data vs. individuals with any missing data. Figures are numbers (percentages) unless stated otherwise Table S7 Comparison of key baseline characteristics and crude incident rates of dementia by exposure status for individuals with complete data vs. individuals with any missing data. Figures are numbers (percentages) unless stated otherwise Table S8 Post hoc analysis with additional adjustment for history of stroke: incidence rates of dementia by treatment and crude and adjusted hazard ratios in people taking angiotensin receptor blockers (ARBs) or angiotensin-converting enzyme inhibitors (ACEIs) Table S9 Post hoc analyses: incidence rates of dementia by treatment and adjusted hazard ratios in people taking angiotensin receptor blockers (ARB) or angiotensin-converting enzyme inhibitors (ACEI), with and without additional adjustment for history of stroke [file bcp0079-0337-sd1.zip › bcp12511-supp-0001-tableS4.docx]

**Supplementary information**

**Table S4 Read codes for dementia subtypes where angiotensin receptor blockers are not expected to have an effect**

| **Read code** | **Read Term** |
| --- | --- |
| E011000 | Korsakov's alcoholic psychosis |
| E011100 | Korsakov's alcoholic psychosis with peripheral neuritis |
| Eu10611 | [X]Korsakov's psychosis, alcohol induced |
| E011200 | Wernicke-Korsakov syndrome |
| E040.11 | Korsakoff's non-alcoholic psychosis |
| F11x600 | Cerebral degeneration due to vitamin B12 deficiency |
| Eu10711 | [X]Alcoholic dementia NOS |
| E012.11 | Alcoholic dementia NOS |
| Eu10712 | [X]Chronic alcoholic brain syndrome |
| E012000 | Chronic alcoholic brain syndrome |
| Eu02400 | [X]Dementia in human immunodef virus [HIV] disease |
| F11x000 | Cerebral degeneration due to alcoholism |
| F11x500 | Cerebral degeneration due to myxoedema |
| F11x700 | Cerebral degeneration due to Jakob - Creutzfeldt disease |
| Eu02100 | [X]Dementia in Creutzfeldt-Jakob disease |
| E012.00 | Other alcoholic dementia |
| Eu03.11 | [X]Korsakov's psychosis, nonalcoholic |
| F11x400 | Cerebral degeneration due to neoplastic disease |
| F11x800 | Cerebral degeneration due to multifocal leucoencephalopathy |
