# Supplementary material for: Angiotensin receptor blockers and risk of dementia: cohort study in UK Clinical Practice Research Datalink
Source: Br J Clin Pharmacol. 2015 Jan 20;79(2):337–50. doi: 10.1111/bcp.12511 (PMC4309639; doi:10.1111/bcp.12511)
Supplement: Supplementary file 1 — Table S1 List of angiotensin-converting enzyme inhibitors included in the study Table S2 List of angiotensin II receptor blockers included in the study Table S3 Read codes representing dementia outcomes of interest in the study Table S4 Read codes for dementia subtypes where angiotensin receptor blockers are not expected to have an effect Table S5 Read codes indicative of pre-existing dementia or cognitive impairment Table S6 Comparison of key baseline characteristics and crude incident rates of dementia for individuals with complete data vs. individuals with any missing data. Figures are numbers (percentages) unless stated otherwise Table S7 Comparison of key baseline characteristics and crude incident rates of dementia by exposure status for individuals with complete data vs. individuals with any missing data. Figures are numbers (percentages) unless stated otherwise Table S8 Post hoc analysis with additional adjustment for history of stroke: incidence rates of dementia by treatment and crude and adjusted hazard ratios in people taking angiotensin receptor blockers (ARBs) or angiotensin-converting enzyme inhibitors (ACEIs) Table S9 Post hoc analyses: incidence rates of dementia by treatment and adjusted hazard ratios in people taking angiotensin receptor blockers (ARB) or angiotensin-converting enzyme inhibitors (ACEI), with and without additional adjustment for history of stroke [file bcp0079-0337-sd1.zip › bcp12511-supp-0001-tableS5.docx]

**Supplementary information**

**Table S5 Read codes indicative of pre-existing dementia or cognitive impairment**

| **Read code** | **Read Term** |
| --- | --- |
| 1461.00 | H/O: dementia |
| 3A...12 | Dementia assessment |
| 6AB..00 | Dementia annual review |
| ZR1K.00 | Alzheimer's disease assessment scale |
| 3AE..00 | Global deterioration scale: assessment of prim deg dementia |
| ZR1K.11 | ADAS - Alzheimer's disease assessment scale |
| F112.00 | Senile degeneration of brain* |
| 9hD0.00 | Excepted from dementia quality indicators: Patient unsuitabl |
| F11yz00 | Other cerebral degeneration NOS* |
| F11y.00 | Other cerebral degeneration* |
| ZR3V.11 | DRS - Clinical dementia rating scale |
| ZR3V.13 | Dementia rating scale |
| 9hD1.00 | Excepted from dementia quality indicators: Informed dissent |
| 9hD..00 | Exception reporting: dementia quality indicators |
| F11xz00 | Cerebral degeneration other disease NOS* |
| ZR3V.00 | Clinical dementia rating scale |
| 9Ou1.00 | Dementia monitoring first letter |
| F11x200 | Cerebral degeneration due to cerebrovascular disease* |
| 66h..00 | Dementia monitoring |
| 3AE3.00 | GDS level 4 - moderate cognitive decline |
| 9Ou5.00 | Dementia monitoring telephone invite |
| ZR3V.12 | CDR - Clinical dementia rating scale |
| 3AE4.00 | GDS level 5 - moderately severe cognitive decline |
| 3AE6.00 | GDS level 7 - very severe cognitive decline |
| 9Ou2.00 | Dementia monitoring second letter |
| 9Ou..00 | Dementia monitoring administration |
| 9Ou3.00 | Dementia monitoring third letter |
| 9Ou4.00 | Dementia monitoring verbal invite |
| 3AE5.00 | GDS level 6 - severe cognitive decline |
| F11x900 | Cerebral degeneration in Parkinson's disease* |
| F11x.00 | Cerebral degeneration in other disease EC* |
| ZR2X.12 | BDRS - Blessed dementia rating scale |
| ZR1T.00 | Arizona battery for communication disorders of dementia |

*These codes were included as these conditions could be associated with cognitive impairment. Thus, patients with these codes prior to index date were also excluded from the analysis.
