# Supplementary material for: Angiotensin receptor blockers and risk of dementia: cohort study in UK Clinical Practice Research Datalink
Source: Br J Clin Pharmacol. 2015 Jan 20;79(2):337–50. doi: 10.1111/bcp.12511 (PMC4309639; doi:10.1111/bcp.12511)
Supplement: Supplementary file 1 — Table S1 List of angiotensin-converting enzyme inhibitors included in the study Table S2 List of angiotensin II receptor blockers included in the study Table S3 Read codes representing dementia outcomes of interest in the study Table S4 Read codes for dementia subtypes where angiotensin receptor blockers are not expected to have an effect Table S5 Read codes indicative of pre-existing dementia or cognitive impairment Table S6 Comparison of key baseline characteristics and crude incident rates of dementia for individuals with complete data vs. individuals with any missing data. Figures are numbers (percentages) unless stated otherwise Table S7 Comparison of key baseline characteristics and crude incident rates of dementia by exposure status for individuals with complete data vs. individuals with any missing data. Figures are numbers (percentages) unless stated otherwise Table S8 Post hoc analysis with additional adjustment for history of stroke: incidence rates of dementia by treatment and crude and adjusted hazard ratios in people taking angiotensin receptor blockers (ARBs) or angiotensin-converting enzyme inhibitors (ACEIs) Table S9 Post hoc analyses: incidence rates of dementia by treatment and adjusted hazard ratios in people taking angiotensin receptor blockers (ARB) or angiotensin-converting enzyme inhibitors (ACEI), with and without additional adjustment for history of stroke [file bcp0079-0337-sd1.zip › bcp12511-supp-0001-tableS8.docx]

Supplementary Information

Table S8 Post-hoc analysis with additional adjustment for history of stroke - incidence rates of dementia by treatment and crude and adjusted hazard ratios in people taking angiotensin receptor blockers (ARBs) or angiotensin converting enzyme inhibitors (ACEIs)

| **ARB exposure** | **Total number**  **of new diagnoses of dementia** | **Total person-time (x10^5^ person-yrs)** | **Incidence rate**  **(per 10^3^ pyrs)** | **Crude HR**  **(95% CI)** | **p value** | **Adjusted HR***  **(95% CI)** | **p value** |
| --- | --- | --- | --- | --- | --- | --- | --- |
| **ARB exposure** |  |  |  |  |  |  |  |
| ACEI use only | 5853 | 14.96 | 3.91 (3.81-4.01) | 1.0 |  | 1.0 |  |
| Ever ARB use | 664 | 1.92 | 3.46 (3.20-3.73) | 0.89 (0.82-0.97) | 0.004 | 0.93 (0.85-1.01) | 0.06 |
| **Age at first prescription (years)** |  |  |  |  |  |  |  |
| 18-54 | 51 | 4.46 | 0.11 (0.09-0.15) | 1.0 |  | 1.0 |  |
| 55-64 | 331 | 4.54 | 0.73 (0.65-0.81) | 6.39 (4.76-8.58) |  | 2.83 (1.87-4.30) |  |
| 65-74 | 1949 | 4.60 | 4.24 (4.06-4.43) | 37.5 (28.38-49.48) |  | 6.03 (3.87-9.40) |  |
| ≥75 | 4186 | 3.28 | 12.77 (12.39-13.16) | 118.72 (90.07-156.49) | <0.001 | 9.42 (5.97-14.86) | <0.001 |
| **Gender** |  |  |  |  |  |  |  |
| Female | 4015 | 8.01 | 5.01 (4.86-5.17) | 1.0 |  | 1.0 |  |
| Male | 2502 | 8.86 | 2.82 (2.71-2.94) | 0.56 (0.54-0.59) | <0.001 | 0.81 (0.77-0.86) | <0.001 |
| **BMI** |  |  |  |  |  |  |  |
| Underweight | 497 | 0.43 | 11.43 (10.47-12.48) | 1.0 |  | 1.0 |  |
| Normal | 2367 | 3.89 | 6.08 (5.84-6.33) | 0.52 (0.48-0.58) |  | 0.74 (0.67-0.81) |  |
| Overweight | 3653 | 12.55 | 2.91(2.82-3.01) | 0.25 (0.23-0.27) | <0.001 | 0.52 (0.47-0.57) | <0.001 |
| **Smoking** |  |  |  |  |  |  |  |
| No | 3728 | 8.78 | 4.25 (4.11-4.39) | 1.0 |  | 1.0 |  |
| Ex | 1995 | 4.96 | 4.02 (3.85-4.20) | 0.96 (0.91-1.01) |  | 1.03 (0.97-1.09) |  |
| Current | 794 | 3.14 | 2.53 (2.36-2.71) | 0.60 (0.56-0.65) | <0.001 | 1.03 (0.95-1.11) | 0.52 |
| **Alcohol** |  |  |  |  |  |  |  |
| No | 1903 | 3.30 | 5.76 (5.11-6.03) | 1.0 |  | 1.0 |  |
| Ex | 227 | 0.40 | 5.67 (4.98-6.46) | 1.00 (0.87-1.15) |  | 1.24 (1.08-1.42) |  |
| Current low | 1648 | 4.00 | 4.12 (3.93-4.32) | 0.71 (0.67-0.76) |  | 0.89 (0.83-0.95) |  |
| Current medium | 73 | 0.35 | 2.11 (1.67-2.65) | 0.36 (0.29-0.46) |  | 0.79 (0.62-1.00) |  |
| Current high | 51 | 0.23 | 2.22 (1.68-2.92) | 0.39 (0.29-0.51) |  | 1.22 (0.92-1.61) |  |
| Current unknown amount | 2615 | 8.60 | 3.04 (2.93-3.16) | 0.53 (0.50-0.56) | <0.001 | 0.85 (0.80-0.90) | <0.001 |
| **Hypertension** |  |  |  |  |  |  |  |
| No | 265 | 0.84 | 3.14 (2.79-3.55) | 1.0 |  | 1.0 |  |
| Yes | 6252 | 16.03 | 3.90 (3.80-4.00) | 1.27 (1.12-1.43) | <0.001 | 0.92 (0.82-1.05) | 0.24 |
| **Heart failure** |  |  |  |  |  |  |  |
| No | 5583 | 15.78 | 3.54 (3.45-3.63) | 1.0 |  | 1.0 |  |
| Yes | 934 | 1.11 | 8.42 (7.90-8.98) | 2.37 (2.22-2.54) | <0.001 | 1.15 (1.07-1.24) | <0.001 |
| **Diabetes** |  |  |  |  |  |  |  |
| No | 4816 | 12.85 | 3.75 (3.64-3.86) | 1.0 |  | 1.0 |  |
| Yes – no metformin/insulin | 992 | 1.91 | 5.19 (4.88-5.53) | 1.40 (1.31-1.50) |  | 1.11 (1.04-1.19) |  |
| Yes - metformin | 525 | 1.50 | 3.50 (3.22- 3.82) | 0.95 (0.87-1.04) |  | 1.19 (1.09-1.31) |  |
| Yes - insulin | 184 | 0.62 | 2.99 (2.58- 3.45) | 0.80 (0.69-0.92) | <0.001 | 1.20 (1.04-1.40) | 0.001 |
| **Statin use** |  |  |  |  |  |  |  |
| No | 4064 | 10.48 | 3.88 (3.76-4.00) | 1.0 |  | 1.0 |  |
| Yes | 2453 | 6.39 | 3.84 (3.69-3.99) | 1.03 (0.98-1.08) | 0.30 | 0.99 (0.94-1.04) | 0.63 |
| **SES** |  |  |  |  |  |  |  |
| I | 1245 | 3.18 | 3.92 (3.71-4.14) | 1.0 |  | 1.0 |  |
| II | 1230 | 3.29 | 3.74 (3.53-3.95) | 0.96 (0.88-1.03) |  | 0.97 (0.89-1.04) |  |
| III | 1321 | 3.24 | 4.07 (3.86-4.30) | 1.05 (0.97-1.13) |  | 1.00 (0.92-1.08) |  |
| IV | 1332 | 3.57 | 3.74 (3.54-3.94) | 0.96 (0.89-1.03) |  | 0.95 (0.87-1.02) |  |
| V | 1389 | 3.60 | 3.86 (3.66-4.07) | 0.99 (0.91-1.06) | 0.13 | 1.09 (1.01-1.18) | 0.002 |
| **Calendar year** |  |  |  |  |  |  |  |
| 1995-1999 | 1412 | 3.62 | 3.90 (3.70-4.10) | 1.0 |  | 1.0 |  |
| 2000-2004 | 3525 | 8.60 | 4.10 (3.97-4.24) | 1.16 (1.09-1.24) |  | 1.14 (1.06-1.22) |  |
| 2005-2010 | 1580 | 4.66 | 3.39 (3.23-3.57) | 1.07 (0.98-1.16) | <0.001 | 1.16 (1.07-1.27) | 0.001 |
| **No. of consultations** |  |  |  |  |  |  |  |
| ≤2 | 224 | 1.11 | 2.01 (1.77-2.30) | 1.0 |  | 1.0 |  |
| 3-5 | 824 | 3.33 | 2.47 (2.31-2.65) | 1.22 (1.06-1.42) |  | 1.05 (0.90-1.22) |  |
| 6-9 | 1843 | 5.25 | 3.51 (3.36-3.68) | 1.74 (1.51-1.99) |  | 1.14 (0.99-1.31) |  |
| 10-14 | 1862 | 4.23 | 4.40 (4.21-4.61) | 2.19 (1.91-2.52) |  | 1.19 (1.04-1.37) |  |
| ≥15 | 1764 | 2.96 | 5.97 (5.70-6.25) | 3.02 (2.63-3.47) | <0.001 | 1.40 (1.21-1.61) | <0.001 |
| **Attained age (time-updated variable) (years)** |  |  |  |  |  |  |  |
| <60 | 65 | 5.01 | 0.13 (0.10-0.17) | 1.0 |  | 1.0 |  |
| ≥60 | 358 | 4.58 | 0.78 (0.70-0.87) | 6.00 (4.61-7.82) |  | 2.18 (1.49-3.18) |  |
| ≥70 | 2154 | 4.53 | 4.75 (4.56-4.96) | 36.47 (28.50-46.68) |  | 6.27(4.19-9.37) |  |
| ≥80 | 3940 | 2.76 | 14.27 (13.83-14.72) | 109.32 (85.55-139.72) | <0.001 | 11.49 (7.59-17.39) | <0.001 |
| **History of stroke** |  |  |  |  |  |  |  |
| No | 5491 | 15.77 | 3.49 (3.40-3.58) | 1.0 |  | 1.0 |  |
| Yes | 1020 | 1.11 | 9.21 (8.67-9.79) | 2.67 (2.50-2.86) | <0.001 | 1.51 (1.41-1.62) | <0.001 |

Pyrs=person years

BMI=body mass index

SES=social economic status

HR=hazard ratio

*Adjusted for age at first prescription, sex, BMI, smoking, alcohol use, diabetes, hypertension, heart failure, statin use, social economic status, calendar year, number of consultations in last 6 months and attained age (time-updated variable) and history of stroke.

Likelihood ratio test used for significance testing
